# Biological and therapeutic effects of ortho-silicic acid and some ortho-silicic acid-releasing compounds: New perspectives for therapy

**DOI:** 10.1186/1743-7075-10-2

**Published:** 2013-01-08

**Authors:** Lela Munjas Jurkić, Ivica Cepanec, Sandra Kraljević Pavelić, Krešimir Pavelić

**Affiliations:** 1 Department of Biotechnology, University of Rijeka, Radmile Matejčić 2, Rijeka, HR-51000, Croatia; 2 PharmaS Ltd, Industrijska cesta 5, Potok, Popovača, HR-44317, Croatia

**Keywords:** Silicon, Orthosilicic acid, Zeolites, Therapeutic and biological effects

## Abstract

Silicon (Si) is the most abundant element present in the Earth's crust besides oxygen. However, the exact biological roles of silicon remain unknown. Moreover, the ortho-silicic acid (H_4_SiO_4_), as a major form of bioavailable silicon for both humans and animals, has not been given adequate attention so far. Silicon has already been associated with bone mineralization, collagen synthesis, skin, hair and nails health atherosclerosis, Alzheimer disease, immune system enhancement, and with some other disorders or pharmacological effects. Beside the ortho-silicic acid and its stabilized formulations such as choline chloride-stabilized ortho-silicic acid and sodium or potassium silicates (e.g. M_2_SiO_3_; M= Na,K), the most important sources that release ortho-silicic acid as a bioavailable form of silicon are: colloidal silicic acid (hydrated silica gel), silica gel (amorphous silicon dioxide), and zeolites. Although all these compounds are characterized by substantial water insolubility, they release small, but significant, equilibrium concentration of ortho-silicic acid (H_4_SiO_4_) in contact with water and physiological fluids. Even though certain pharmacological effects of these compounds might be attributed to specific structural characteristics that result in profound adsorption and absorption properties, they all exhibit similar pharmacological profiles readily comparable to ortho-silicic acid effects. The most unusual ortho-silicic acid-releasing agents are certain types of zeolites, a class of aluminosilicates with well described ion(cation)-exchange properties. Numerous biological activities of some types of zeolites documented so far might probably be attributable to the ortho-silicic acid-releasing property. In this review, we therefore discuss biological and potential therapeutic effects of ortho-silicic acid and ortho-silicic acid -releasing silicon compounds as its major natural sources.

## Introduction

Silicon (Si) is the most abundant element (27.2%) present in the earth's crust following oxygen (45.5%) [1]. Silicon is known for a number of important chemical and physical properties, *i.e.* semiconductor property that are used in various scientific and technical applications. These Si features, along with structural complexity of its compounds, have attracted researchers from the earliest times [2]. In particular, silicon dioxide or silica (SiO_2_) is the most studied chemical compound following water, and the most important Si-containing inorganic substance [1]. Formally, silica (SiO_2_) is a silicic acid anhydride of monomeric ortho-silicic acid (H_4_SiO_4_), which is water soluble and stable in highly diluted aqueous solutions. Moreover, several “lower” hydrated forms of ortho-silicic acid exist in aqueous solutions as well including meta-silicic acid (H_2_SiO_3_ or lower oligomers like di-silicic (H_2_Si_2_O_5_) and tri-silicic acids (H_2_Si_3_O_7_) including their hydrated forms pentahydro-silicic (H_10_Si_2_O_9_), and pyro-silicic acids (H_6_Si_2_O_7_) [1]. These are water soluble, formed in reversible equilibrium reactions from H_4_SiO_4_ and stable in diluted aqueous solutions. During a prolonged storage period, at increased concentration or in an acidic environment, these low molecular silicic acids undergo further condensation by cross-linking and dehydration. This process results in formation of poly-silicic acids chains of variable composition [SiO_x_(OH)_4-2x_ and complex structure [1]. The end product is a jelly-like precipitate, namely hydrated silica (SiO_2_·xH_2_O; often referred as “colloidal silicic acid” or “hydrated silica gel”). Further condensation follows which is accompanied by dehydration yielding less hydrated silicon dioxide (SiO_2_) phases, also known as “silica gel” or “amorphous silicon dioxide”.

**Figure 1:**
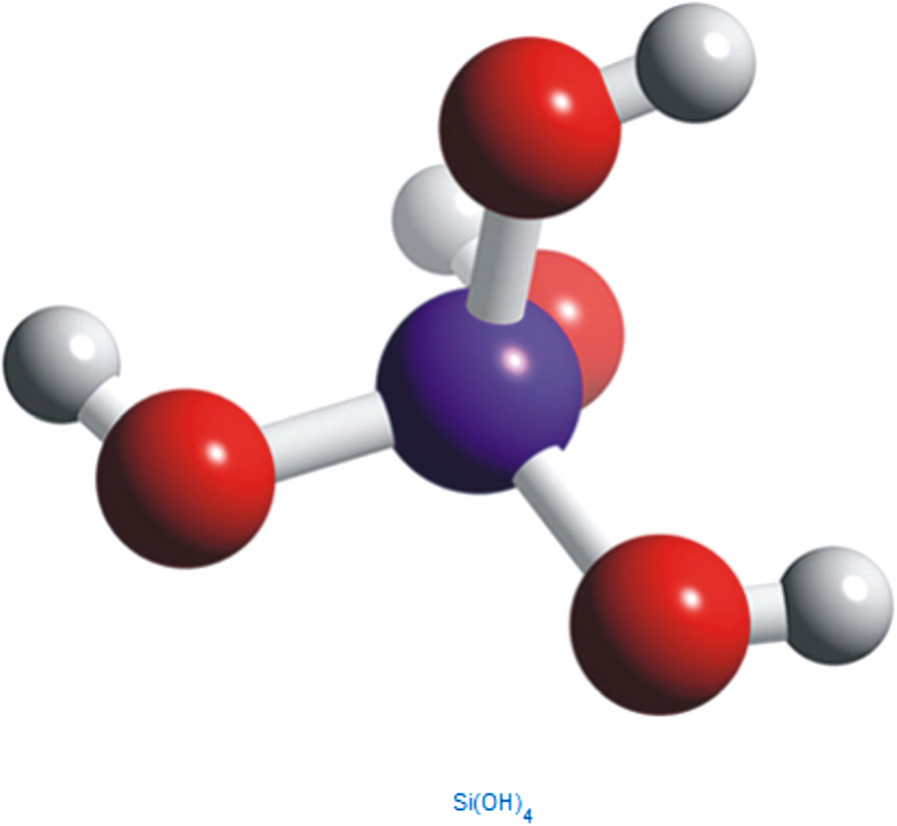
Chemical 3D structure of Si(OH)_4_.

**Figure 2:**
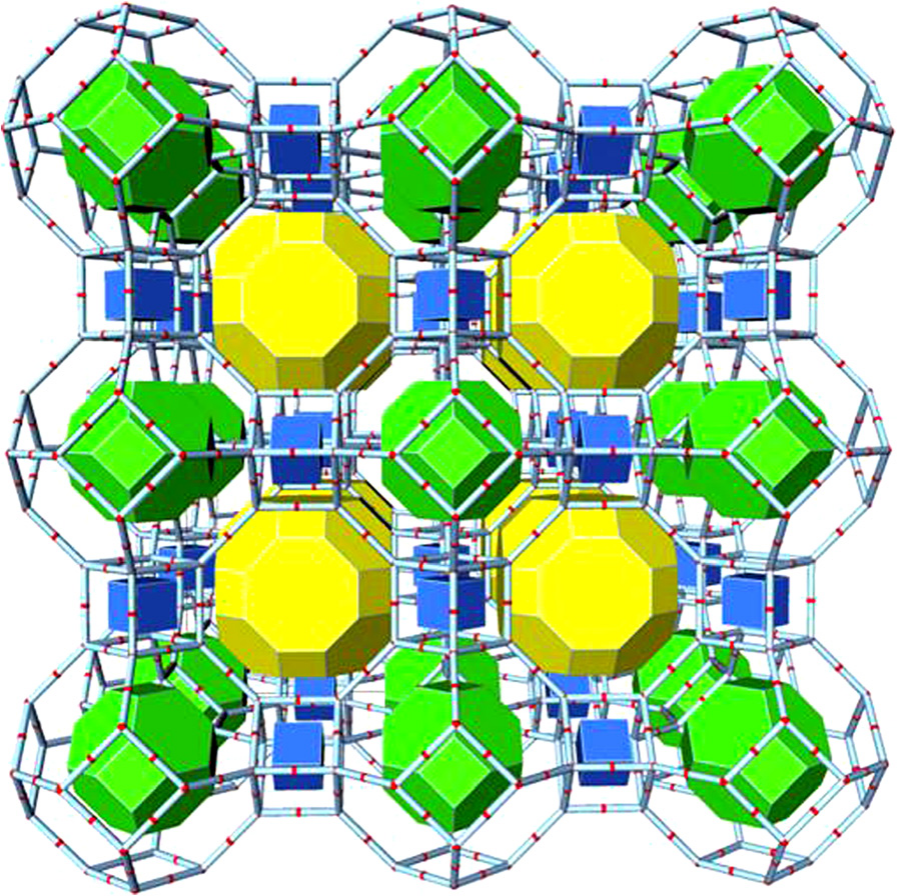
**Zeolite A structure: an assembly of framework's cages (tiles).** Centre of a tile is the centre of a void in the framework. Voids are connected with adjacent ones through the large "windows" which are faces of tiles.

**Figure 3:**
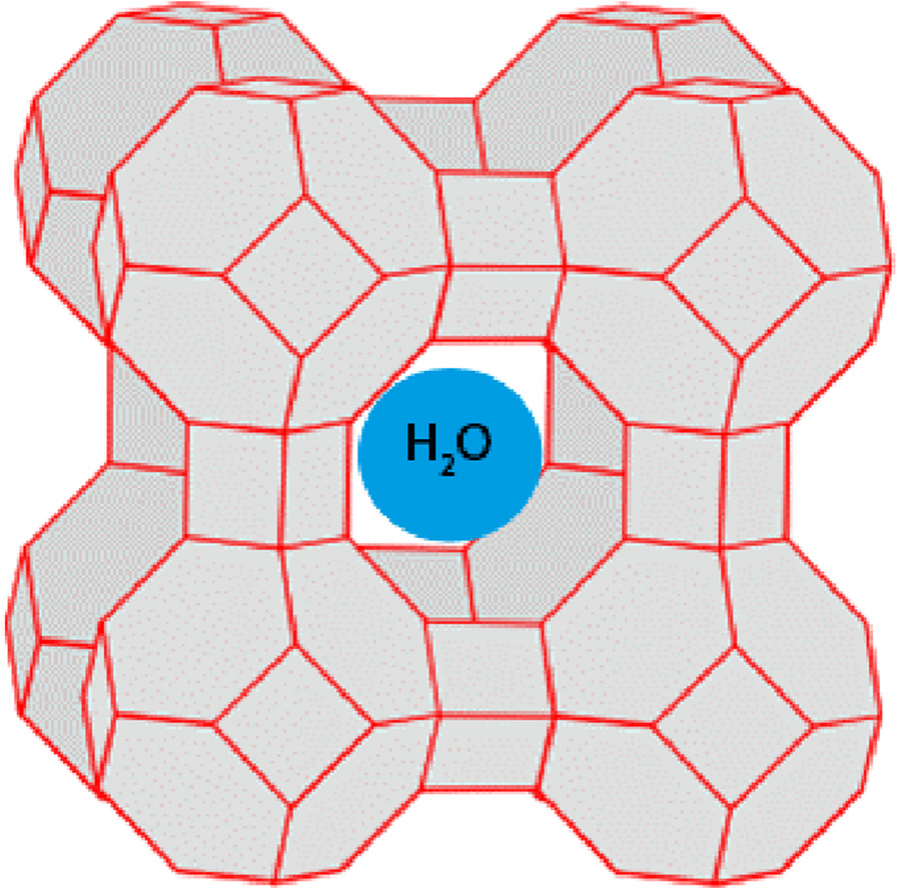
Microporous crystal structure of clinoptilolite.

Lower molecular forms, especially the ortho-silicic acid (H_4_SiO_4_; Figure 1), play a crucial role in delivering silicon to the living organisms’ cells and thus represent major sources of silicon for both humans and animals. Most of the silica in aqueous systems and oceans is available in the form of H_4_SiO_4_, which makes it an important compound in environmental silicon-chemistry and biology [3]. In this paper, we critically review the most recent findings on biological effects of Si and ortho-silicic acid on animals and human beings. Moreover, we propose that previously observed positive biological effects of various colloidal silicic acids (various hydrated silica gels) as well as some zeolites [4-6], e.g. zeolite A (Figure 2) and clinoptilolite (Figure 3), might be, at least partially, ascribed to the ortho-silicic acid-releasing property.

Silicon represents the third most abundant trace element in the human body [7,8]. For example, it is present in 1–10 parts-per-million (ppm) in hair [9], nails [10], in the cornfield epidermis, and in the epicuticle of hair [11,12]. Silicon is naturally present in food as a silicon dioxide (SiO_2_), free ortho-silicic acid (H_4_SiO_4_), silicic acids bounded to certain nutrients, and in the silicate form. Although silicon is a life-important micronutrient mineral, in our opinion it has not received adequate attention. Considering the abundance of silicon, both in the nature and humans, it is expected that it should play an important role in human and animal health.

## Silicon bioavailability and consumption

Presently, many biological roles of silicon remain unknown [13]. Consequently, the recommended daily silicon intake (RDI) has not yet been set [13,14]. Considering the risk assessment of amorphous silicon dioxide as common silicon source (e.g. food additive E551), the safe upper intake level (UIL) may be estimated as 700 mg/day for adults, that is the equivalent to 12 mg silicon/kg bw/day for a 60 kg adult [15]. These numbers refer to the amorphous silicon dioxide form and only small amounts of silicon (as H_4_SiO_4_) are actually released in the gastrointestinal (GI) tract and subsequently absorbed in the systemic circulation. Due to lack of data, it is difficult to set a recommended upper intake level for silicon. Moreover, little information on the intake of dietary silicon by humans is available. A mean intake of daily silicon has been reported in Finland [16], (29 mg silicon/day) and in a typical British diet (20–50 mg silicon/day) [17-19]. This corresponds to 0.3-0.8 mg/silicon/kg bw/day for a 60 kg person. These data are in the same range as the estimated mean intakes of silicon in the USA (30 and 33 mg silicon/day in men, and 24 and 25 mg silicon/day in women, respectively) [8]. Silicon intake decreases with age to less than 20 mg silicon/day (18.6 ± 4.6 mg silicon/day for elderly British woman in an unrelated randomised controlled intervention study) [20].

Generally, silicon is abundantly present in foods derived from plants such as: cereals, oats, barley, white wheat flour, and polished rice. In contrast, silicon levels are lower in animal foods including meat or dairy products. Furthermore, silicon is present in drinking waters, mineral waters, and in beer as well [17]. However, Jugdaohsingh et al. [21] raised some doubt on utilisation of silicon from drinking water in an animal rat study as no significant differences were found in the silicon bone concentration when the drinking water was supplemented with silicon in the ortho-silicic acid form. Indeed, the major sources of silicon in the typical Western hemisphere diet comes from cereals (30%), followed by fruits, beverages and vegetables, which altogether comprise around 75% of total silicon intake [20]. Even though plant food contains high levels of silicon, its bioavailability from these sources is questionable, due to poor solubility of actual silicon forms present in these foods [18,19,22]. Efficient absorption in the GI tract would require their breakdown to soluble species such as ortho-silicic acid, present in drinking and mineral waters in the range of 2 to 5 mg silicon/L [23] and in beer ranging from 9 to 39 mg silicon/L [18,24]. Absorption studies indicate that the ortho-silicic acid is a main readily bioavailable source of silicon for humans, whereas its higher polymers are not of significant absorbability [25]. In a placebo-controlled study on eight volunteers, Jugdaohsingh et al. [25] showed that 53% of administered ortho-silicic acid is excreted in the urine, whereas the ingestion of polymeric silicic acid causes only a marginal increase of silicon in the urine. This result substantiates the statement that polymeric silicic acids and amorphous silicon dioxide are of poor bioavailability.

Besides the ortho-silicic acid, water soluble silicates are bioavailable silicon forms as well. For instance, pharmaceutically acceptable alkali metals silicates (M_2_SiO_3_; M= Na, K) in adequately diluted aqueous solutions, release ortho-silicic acid (H_4_SiO_4_) upon contact with stomach hydrochloric acid (HCl). Popplewell et al. [26] employed a tracer dose of radiolabelled ammonium silicate to measure total uptake and urine excretion. Their results revealed that 36% of ingested dose was absorbed and completely excreted in urine within 48h. However, elimination occurred in two steps where the major dose (90%) has been excreted within the first 2.7 hours. They suggested that excess silicon is eliminated from the body through two distinct processes, differing significantly in the duration. The ‘slower process’ is thought to include the intracellular uptake and release of silicon, whilst the ‘faster process’ probably includes retention of silicon in the extracellular fluids [26]. These data report on increased silicon levels in serum upon consumption of silicon-rich food [7,27], showing that at least some silicon is available from food as well. Indeed, selective silicon deprivation in rats showed a significant drop of urinary silicon excretion and fasting silicon serum concentration, suggesting that the rats actively regulate silicon levels *via* urinary conservation, perhaps through renal re-absorption [21]. Most of silicon present in the serum is filtered by the kidney [7,28] suggesting the kidney as its major excretion route; silicon levels in serum correlate with those in urine. However, it is still not clear how and if the body can efficiently retain adequate doses of silicon.

In concentrated solutions, ortho-silicic acid (H_4_SiO_4_) has to be stabilized to avoid its polymerization into poly-silicic acids and eventually into silica gel, resulting in a decreased silicon bioavailability. This issue has been solved in the field of pharmaceutical technology by use of choline chloride in aqueous glycerol solution. This resulted in development of a liquid formulation known as choline-stabilized ortho-silicic acid (ch-OSA). Choline chloride-stabilized ortho-silicic acid is not a new chemical entity of ortho-silicic acid, but a complex of H_4_SiO_4_ and choline chloride formed by several possible hydrogen bonds between these two compounds. Subsequently, from the standpoint of nutrition and pharmacology, the effects of ch-OSA must involve effects of both H_4_SiO_4_ and choline chloride rather than a new chemical entity. Due to a possible impact of choline chloride on the chemical stability of H_4_SiO_4_, certain specific biological effects different from those of a pure ortho-silicic acid or its immediate releasing compounds (e.g. sodium silicate), must be taken in account. Ch-OSA has been approved for human consumption and is known to be non-toxic. Its lethal doses (LD) exceed 5000 mg/kg bw in humans [29] and 6640 mg/kg bw in animals [30]. The ch-OSA represents the most bioavailable source of silicon [22,29]. Moreover, in a randomized placebo-controlled study [29], the bioavailability of ch-OSA during maternal transfer to the offspring was investigated in a supplementation study with pigs. The authors correlated significantly higher silicon concentrations in the serum of weanling piglets from supplemented sows and maternal transfer of absorbed silicon between sows and their offspring during lactation with high bioavailability of silicon from ch-OSA. Importantly, highly bioavailable silicon from ch-OSA did not altered calcium, phosphorus and magnesium levels in blood.

## Therapeutic and biological effects of ortho-silicic acid and certain ortho-silicic acid-releasing compounds

It was reported that silicon is connected with bone mineralization and osteoporosis [31], collagen synthesis and ageing of skin [11], condition of hair and nails [32], atherosclerosis [33,34], Alzheimer disease [9,35,36], as well as with other biological effects and disorders. Trace minerals are known to generally play a vital role in the human body homeostasis [37] and the serum levels of silicon are similar to other trace elements, *i.e.* of iron, copper, and zinc [38]. Silicon is excreted through the urine in similar orders of magnitude as calcium. Some researches claim that silicon does not act as a protein-bounding element in plasma and is believed to exist almost entirely as un-dissociated monomeric ortho-silicic acid [28]. While early analyses showed that serum contains 50–60 μg silicon/dL [38,39], more recent analyses indicate that human serum contains 11–25 μg silicon/dL, or levels ranging between 24 and 31 μg/dL (8.5 and 11.1 μmol/L), detected by absorption spectrometry in large population groups [40]. Interestingly, pregnant women had very low serum silicon concentrations (3.3-4.3 μg/dL) in comparison with infants that have high concentrations between 34 and 69 μg/dL [27,41]. Moreover, silicon concentrations in serum showed a statistically significant age and sex dependency, as it seems that silicon concentrations decrease with age, especially in woman [40].

Biological importance of silicon might be analysed in the context of its bio-distribution in the body. For example, the highest silicon concentration has been measured in connective tissues, especially in the aorta, tracheas, bone, and skin. Low levels of silicon in the form of ortho-silicic acid [42-44] may be found in liver, heart, muscle, and lung [45]. It is therefore plausible to assume that observed decrease of silicon concentration in the ageing population may be linked to several degenerative disorders, including atherosclerosis. Supplementation of the regular diet with bioavailable forms of silicon may therefore have a therapeutic potential including prevention of degenerative processes. Several experiments have already confirmed this hypothesis. For example, in a controlled animal study, spontaneously hypertensive rats had lower blood pressure upon supplementation with soluble silicon [44], whilst silicon deficiency in animals has been found to be connected with bone defects and impaired synthesis of connective tissue compounds, such as collagen and glycosaminoglycans [46-48]. It is therefore reasonable to assume that silicon deficiency or lower bioavailability may be linked to problems with bone structure and collagen production. Moreover, silicon was shown to be uniquely localized in active growth areas in young bones of animals where a close relationship between silicon concentration and the degree of mineralization has been assessed [46,49]. Studies confirmed the essential role of silicon in the growth and skeletal development of chicks that during silicon deprivation showed significantly retarded skeletal development [50]. Experimental silicon deprivation in rats [51-53] and chicks [46,47] demonstrated striking effects on skeletal growth and bone metabolism as well. On the other hand, the controlled animal study of Jugdaohsingh et al. [21] showed no profound effects of a silicon-deficient diet on the bone growth and skeletal development in rats. Silicon concentrations in the tibia and soft tissues did not differ from those in rats on a silicon-deficient diet where the silicon was supplemented in drinking water. Nevertheless, silicon levels in tibia were much lower compared to the reference group fed by a silicon rich diet. Body and bone lengths were also found to be lower in comparison with the reference group, while reduction in bone growth plate thickness was found in silicon deprived rats [21].

Moreover, Reffit et al. [54] found that ortho-silicic acid stimulates collagen type 1 synthesis in human osteoblast-like cells and skin fibroblasts and enhances osteoblastic differentiation in the MG-63 cells *in vitro*. Ortho-silicic acid did not alter collagen type 1 gene expression, but it modulated the activity of prolyl hydroxylase, an enzyme involved in the production of collagen [55]. Similarly, Schütze et al. [56] reported that the zeolite A stimulated DNA synthesis in osteoblasts and inhibited osteoclast-mediated bone resorption *in vitro*. This is probably attributable to the ortho-silicic acid-releasing property of zeolite A.

The mechanism underlying observed biological effects of silicon may probably be ascribed to its interrelationships with other elements present in the body such as molybdenum [57] aluminium [9,35,58,59], and calcium [46,49,50]. For instance, it was proven that silicon levels are strongly affected by molybdenum intake, and *vice versa*[59]. Furthermore, silicon accelerates the rate of bone mineralization and calcification as shown in controlled animal studies, in a similar manner that was demonstrated for vitamin D [11,50]. It is well known that vitamin D increases the rate of bone mineralization and bone formation [60], and that its deficiency leads to less mature bone development. Vitamin D is known to be important in calcium metabolism, but silicon-deficient cockerels’ skulls in a controlled animal study showed lower calcification and collagen levels irrespective of the vitamin D dietary levels suggesting a vitamin D-independent mechanism of action [61]. Jugdaohsingh et al. [21] found that silicon supplementation in drinking water did not significantly altered silicon concentrations in bones and suggested that some other nutritional co-factor is required for maximal silicon uptake into bone and that this co-factor was absent in rats fed with a low-silicon diet compared to the reference group fed by a silicon-rich diet. They suggested vitamin K as such co-factor, which is important in bone mineralisation through carboxylation of osteocalcin, and whose deficiency might influence incorporation of minerals such as silicon in the bones.

### Osteoporosis

Osteoporosis is among leading causes of morbidity and mortality worldwide [62]. It is defined as a progressive skeletal disorder, characterised by low bone mass (osteopenia) and micro-architectural deterioration [63]. Interestingly, the administration of silicon in a controlled clinical study induced a significant increase in femoral bone mineral density in osteoporotic women [31]. Direct relationship between silicon content and bone formation has been shown by Moukarzel et al. [64]. They found a correlation between decreased silicon concentrations in total parenterally fed infants with a decreased bone mineral content. This was the first observation of a possible dietary deficiency of silicon in humans. A randomized controlled animal study on aged ovariectomized rats revealed that long-term preventive treatment with ch-OSA prevented partial femoral bone loss and had a positive effect on the bone turnover [65]. Dietary silicon is associated with postmenopausal bone turnover and bone mineral density at the women's age when the risk of osteoporosis increases. Moreover, in a cohort study on 3198 middle-aged woman (50–62 years) it was shown that silicon interacts with the oestrogen status on bone mineral density, suggesting that oestrogen status is important for the silicon metabolism in bone health [66].

### Skin and hair

Typical sign of ageing skin is fall off of silicon and hyaluronic acid levels in connective tissues. This results in loss of moisture and elasticity in the skin. Appearance of hair and nails can also be affected by lower silicon levels, since they are basically composed of keratin proteins. As previously discussed, ortho-silicic acid may stimulate collagen production and connective tissue function and repair. For example, Barel et al. [67] conducted experiments on females, aged between 40–65 years, with clear clinical signs of photo-ageing of facial skin. Their randomized double-blinded placebo-controlled study illustrates positive effects of ch-OSA taken as an oral supplement on skin micro relief and skin anisotropy in woman with photo-aged skin. Skin roughness and the difference in longitudinal and lateral shear propagation time decreased in the ch-OSA group, suggesting improvement in isotropy of the skin. In addition, ch-OSA intake positively affected the brittleness of hair and nails. Oral supplementation with ch-OSA had positive effects on hair morphology and tensile strengths, as shown in a randomized placebo-controlled double blind study by Wickett et al. [68].

### Alzheimer disease

Aluminium (as Al^3+^ ion) is a well-known neurotoxin. Aluminium salts may accelerate oxidative damage of biomolecules. Importantly, it has been detected in neurons bearing neurofibrillary tangles in Alzheimer's and Parkinson's disease with dementia as shown in controlled studies [69,70]. Amorphous aluminosilicates have been found at the core of senile plaques in Alzheimer's disease [69,71], and have consequently been implicated as one of the possible causal factors that contribute to Alzheimer’s disease. Since aluminosilicates are water insoluble compounds, the transport path to the brain is still not well understood. By reducing the bioavailability of aluminium, it may be possible to limit its neurotoxicity. Consumption of moderately high amounts of beer in humans and ortho-silicic acid in animals has shown to reduce aluminium uptake from the digestive tract and slow down the accumulation of this metal in the brain tissue [36,72]. Silicic acid has also been found to induce down-regulation of endogenous antioxidant enzymes associated with aluminium administration and to normalize tumour necrosis factor alpha (TNFα) mRNA expression [35]. Although the effect of silicic acid on aluminium absorption and excretion from human body produced conflicting results so far as shown in an open-label clinical study [7], in a controlled clinical study it was shown that silicic acid substantially reduces aluminium bioavailability to humans [73]. In fact, it was already found that silicon reduces the aluminium toxicity and absorption in some plants and animals that belong to different biological systems [74-76]. This is possible as silicon competes with aluminium in biological systems such as fresh water, as suggested by Birchall and Chappell study perfomed on the geochemical ground [77], and later confirmed by Taylor et al. in randomized double blind study [78]. They found that soft water contains less silicic acid and more aluminium, while hard waters contain more silicic acid and less aluminium.

Removal of aluminium from the body and its reduced absorption by simultaneous administration of silicic acid was tested and proven by Exley et al. in controlled clinical study [59]. They showed reduced urinary excretion of aluminium along with unaltered urinary excretion of trace elements such as iron in persons to whom silicic acid-rich mineral water was administered. Moreover, they documented that regular drinking of a silicon-rich mineral water during a period of 3 months significantly reduced the body burden of aluminium. Similar results were obtained by Davenward et al. [79] who showed that silicon-rich mineral waters can be used as a non-invasive method to reduce the body burden of aluminium in both Alzheimer's patients and control group by facilitating the removal of aluminium *via* the urine without any concomitant effect. They also showed clinically relevant improvements of cognitive performances in at least 3 out of 15 individuals with Alzheimer disease. This implies a possible use of ortho-silicic acid as long-term non-invasive therapy for reduction of aluminium in Alzheimer's disease patients. The mechanism through which aluminium bioavailability reduction occurs involves interaction between aluminium species and ortho-silicic acid where highly insoluble hydroxyaluminosilicates (HAS) forms are produced [77,80]. This process makes aluminium unavailable for absorption.

### Immunostimulatory effects

Quartz as a form of crystalline silicon dioxide has been connected with severe negative biological effects. However, in controlled studies on mouse and rats it was shown that sub-chronic and short-term exposure to this compound can actually have beneficial effects on respiratory defence mechanisms by stimulating immune system through the increase of neutrophils, T lymphocytes and NK cells. It also activates phagocytes and consequently additional ROS production [81-83] which can help the pulmonary clearance of infectious agents. In rats, crystalline silica caused proliferation and activation of CD8^+^ T cells and, to a lesser amount, of CD4^+^ T cells.

Recently, an “anionic alkali mineral complex” Barodon® has shown immunostimulatory effects in horses [84], pigs [85] and other animals. Barodon® is a mixture of sodium silicate (M_2_SiO_3_, M= Na,K) and certain metal salts in an alkaline solution (pH= 13.5), where sodium-silicate (sodium water glass) represents 60% of the total content. In a placebo-controlled experiment in pigs, the immunostimulatory effect of Barodon® was assessed by measurement of proliferation and activation of porcine immune cells, especially CD4^+^ CD8^+^ double-positive (dpp) T lymphocytes in peripheral blood and in the secondary lymphoid organ [85]. As this type of T lymphocyte cells are characterized by a specific memory cell marker CD29, they may play a role during activation of secondary immune responses as shown in a cross-sectional and longitudinal study on pigs [86]. Moreover, Barodon® acted mainly on the lymphoid organs, implying a role in antigenic stimulation of immune tissues [85]. Barodon® induced increased levels of MHC-II lymphocytes and non-T/non-B (N) cells as well along with increased stimulatory mitogen activity including the activity of PHA, concanavalin A, and pokeweed mitogen [85,87]. In a placebo-controlled experiment on pigs, it was shown that this mineral complex exerts an adjuvant effect with hog cholera and Actinobacillus pleuropneumoniae vaccines by increasing the antibody titres and immune cell proportions [88]. Moreover, Barodon® showed nonspecific immunostimulating effects in racing horses and higher phagocytic activity against *Staphylococcus equi* subsp. *equi* and *Staphylococcus aureus* as well in a controlled study [84]. Administration of Barodon® in horse herds reduced many clinical complications, including stress-induced respiratory disease, suggesting activation of immune cell populations similarly to the treatment with inactivated *Propionibacterium acnes*[89,90]. The exact mechanism of Barodon® immunostimulatory effect is not known, although it has been suggested that sodium silicate, the main mineral ingredient, might be responsible for the observed immune-enhancing properties. Indeed, sodium silicate is known to decompose quantitatively into bioavailable ortho-silicic acid (H_4_SiO_4_) in the acidic gastric juice (HCl), and as such being absorbed in the body. In this manner, presumably all observed pharmacological effects of Barodon® are actually originated from the ortho-silicic acid.

Pure sodium metasilicate (Na_2_SiO_3_) also bears immunostimulatory effects and acts as a potent mitochondria activator [91]. Dietary silicon in the form of sodium metasilicate activates formation of ammonia by elevating mitochondrial oxygen utilisation as shown in a controlled animal experiment [91]. These findings further corroborate the hypothesis that sodium silicate might be responsible for immunostimulatory effects of Barodon®. Once again, the pharmacologically active species was ortho-silicic acid released upon the action of stomach hydrochlorid acid on sodium metasilicate.

## Zeolites as a source of ortho-silicic acid

Zeolites are a class of aluminosilicates of general formula (Mn^+^)_x/n_[(AlO_2_)_x_(SiO_2_)_y_·mH_2_O, wherein M represents a positively charged metal ion such as sodium (Na^+^), potassium (K^+^), magnesium (Mg^2+^), or calcium (Ca^2+^). Zeolites are crystalline aluminosilicates with open 3D framework structures built of SiO_4_ and AlO_4_ tetrahedra linked to each other by sharing all the oxygen atoms to form regular intra-crystalline cavities and channels of molecular dimensions [92]. The positively charged metal ions (e.g. Na^+^, K^+^, Ca^2+^, Mg^2+^) are positioned in these cavities of aluminosilicate skeleton which are termed as micro- (2–20 Å), meso- (20–50 Å), and macro-(50–100 Å) -pores. These ions are readily exchangeable in contact with aqueous solution of other positively charged ions (e.g. heavy metal ions like Hg^2+^). This structural characteristic of zeolites is the base of their ion (cation)-exchange property [93].

**Figure 4:**
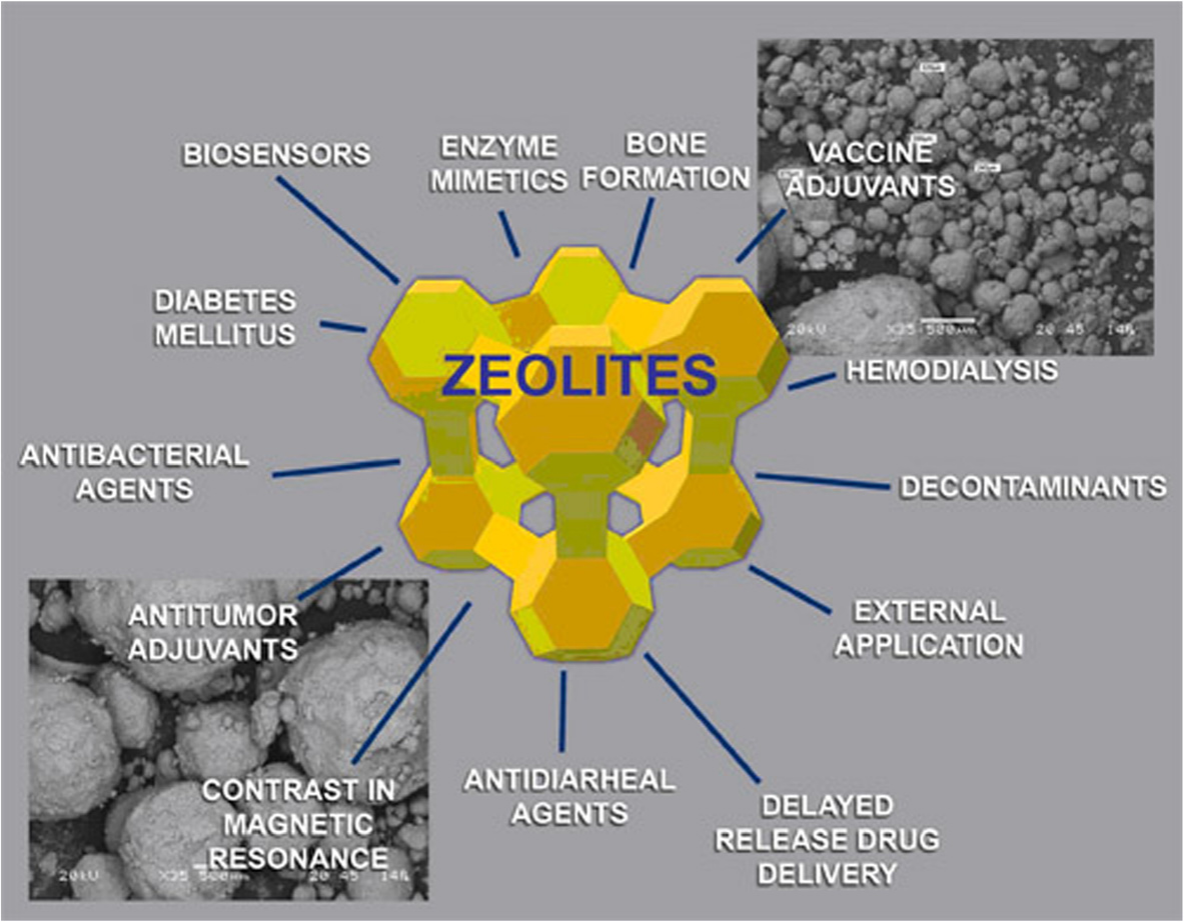
**Biomedical application of zeolites (adopted from: K Pavelic, M Colic, B Subotic.** In: Studies in Surface Science and Catalysis, Vol. 135. Amsterdam: Elsevier, 2001, p 170).

At present, 191 unique zeolite frameworks have been identified [94], while over 40 naturally occurring zeolite frameworks have been described. Zeolites have been widely employed in chemical and food industries, agriculture, and environmental technologies as adsorbents, absorbents, adsorbent filter-aids, ion-exchangers, catalysts, active cosmetic and pharmaceutical ingredients, soil improvers, etc. [95-103]. Besides, zeolites exhibit a number of interesting biological activities [5,104,105] (Figure 4). For example, nontoxic natural zeolite clinoptilolite affects tumour cells proliferation *in vitro* and might act as an adjuvant in cancer therapy [105]. Katic et al. [106] confirmed that clinoptilolite influences cell viability, cell division, and cellular stress response that results in antiproliferative effect and apoptosis induction *in vitro*. Obtained results demonstrated that clinoptilolite biological effect on tumour cells growth inhibition might be a consequence of adsorptive and ion-exchange characteristics that cause adsorption of some serum components by clinoptilolite [106]. Similarly, clinoptilolite showed antiviral effects *in vitro* and a potential in antiviral therapy either for local skin application against herpesvirus infections or oral treatment of adenovirus or enterovirus infections [107]. The antiviral mechanism is probably non-specific and is based on adsorption of viral particles on external cavities at the clinoptilolite surface rather than a consequence of ion-exchange properties.

Each zeolite particle acts like a large inorganic molecule and acts as a molecular sieve with a potential in molecular medicine in molecular medicine. Their pores are indeed, rather small (less than 2 nm to 50 nm) [108], and these structural similarities between the cages of zeolites and binding sites of enzymes resulted in development of zeolite structures that mimic enzyme functions [108], e.g. haemoglobin, cytochrome P450 or iron-sulphur proteins [109].

**Figure 5:**
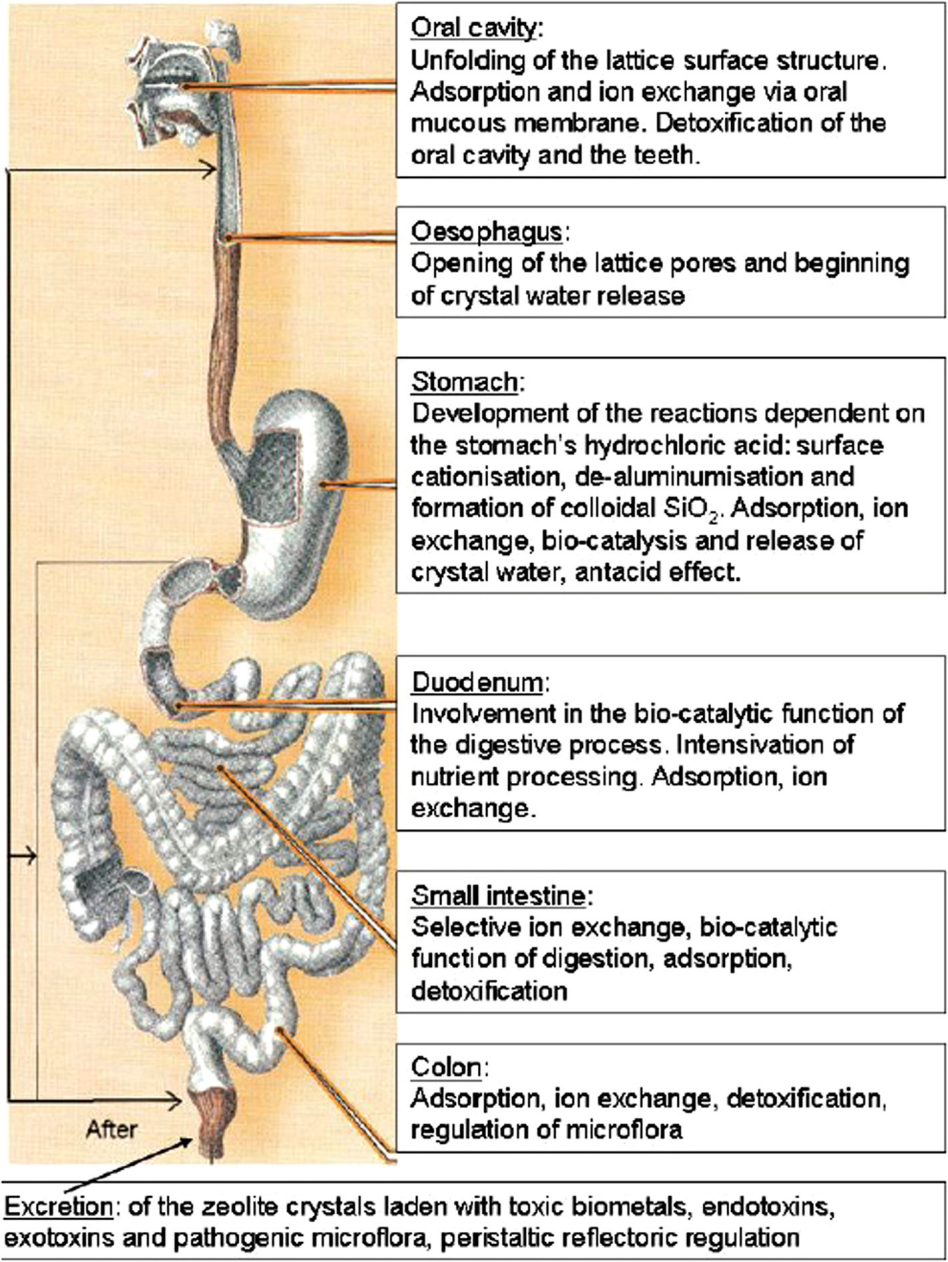
**Structural and biochemical changes of zeolites in the digestive system (by courtesy from Application of natural zeolites in medicine and cosmetology – ZEOMEDCOS.***SWB, Baku-London, 2010)*.

**Figure 6:**
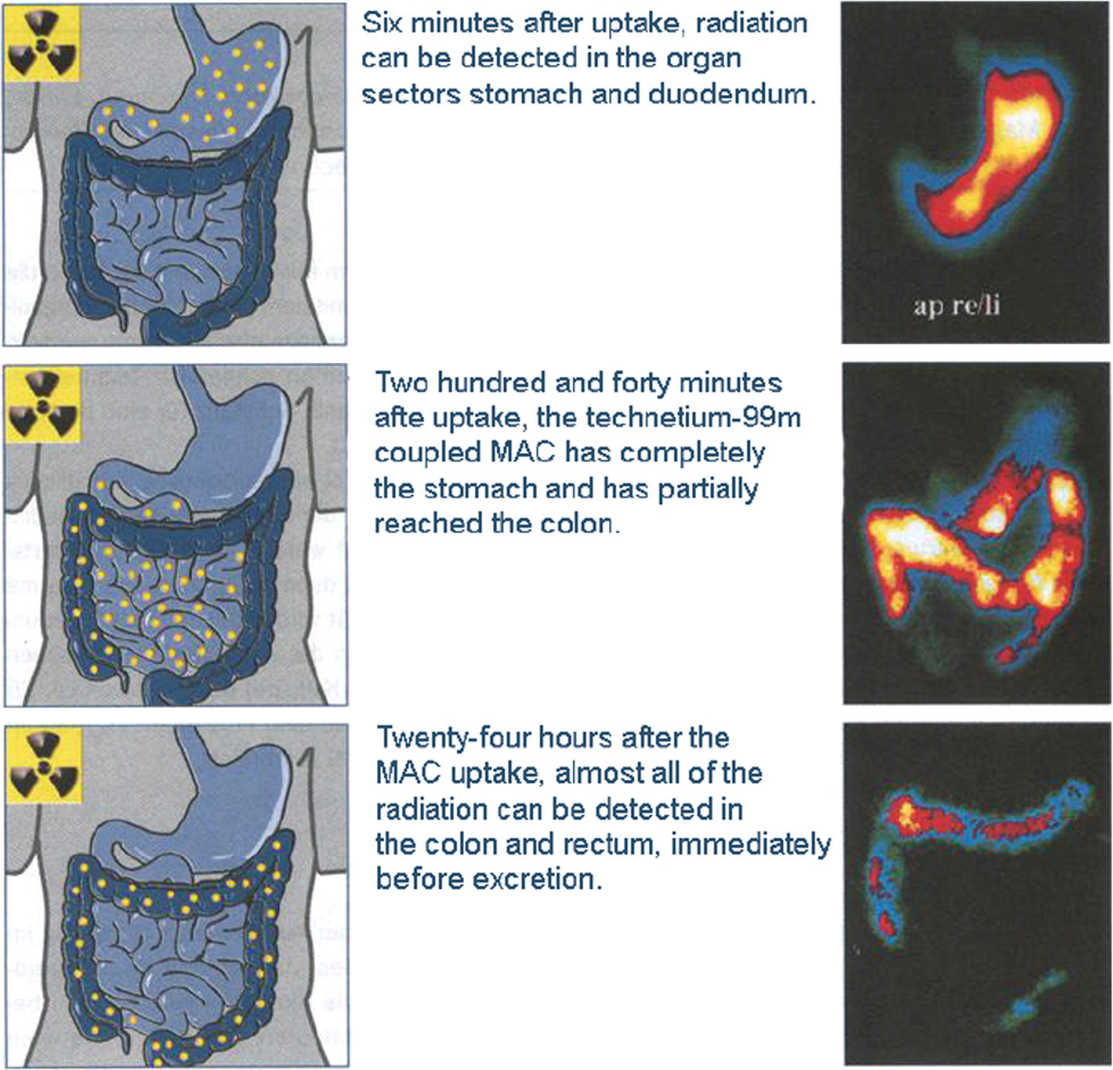
**The fate of isotope labelled activated clinoptilolite-zeolite in the gastro-intestinal tract (by courtesy from Application of natural zeolites in medicine and cosmetology – ZEOMEDCOS.***SWB, Baku-London, 2010)*.

Important data on biological zeolites fate (Figure 5) and effects *in vivo* have been widely reported so far in the scientific literature. For example, it was shown that zeolites bear detoxifying and decontaminant properties when added to animal diets, reducing levels of heavy metals (e.g. lead, mercury, and cadmium) and various organic pollutants, *i.e.* radionuclides (Figure 6) and antibiotics [108]. Furthermore, zeolites have been successfully utilized for haemodialysis, for cartridges in haemoperfusions, for wound healing, and surgical incisions [108]. For instance, QuikClot and Zeomic formulations are already being marketed for haemorrhage control [110] and dental treatment [5], respectively.

Several toxicological studies proved that certain natural zeolite, e.g. clinoptiolite are non-toxic and completely safe for use in human and veterinary medicine [105]. *In vitro* and *in vivo* controlled animal studies have shown that clinoptilolite is an inert substance that may cause, in some instances, only moderate but not progressive fibrosis or mesothelioma [111]. This effect might be attributed to side-substances present in natural zeolites, e.g. silica or clay aluminosilicates [112]. It should be also stated that some zeolites might be extremely dangerous for human health and exert negative biological effects. For example, erionite, a fibrous type of natural zeolite, causes a high incidence of mesotheliomas and fibrosis in humans and experimental animals [113].

Animal studies have also shown the possibility of zeolite A (sodium aluminosilicate) as a viable source of silicon [4,6,114]. The latter is one of known zeolites that breaks down into bioavailable ortho-silicic acid (H_4_SiO_4_) in the digestive system. This property arises from the structure of zeolite A which is characterized by the same number of aluminium and silicon atoms in zeolite A [115]. Zeolite A is hydrolysed at low pH (stomach hydrochloric acid) into ortho-silicic acid (H_4_SiO_4_) and aluminium ions (Al^3+^). These are combined back to the amorphous aluminosilicate. Such process readily provides additional source of bioavailable silicon to the organism [114,116]. Indeed, randomized placebo-controlled studies on dogs [114] proved that silicon is absorbed upon oral administration of zeolite A. Comparable results have been obtained in a randomized placebo-controlled research on horses as well [6]. Addition of zeolite A to the diet of young racing quarter horses have resulted in decreased skeletal injury rates and better training performance [117]. However, increased bone formation was found in randomized controlled studies on broodmare horses [118], but not in yearling horses [119]. Food supplementation with zeolite A in calves showed no changes in bone architecture or mechanical properties [120]. However, in a controlled study Turner et al. [120] showed increased aluminium content in the bone and cartilage of zeolite A-fed calves which is an important safety issue for the zeolite A therapeutic usage.

## Conclusion

In conclusion, we believe that ortho-silicic acid (H_4_SiO_4_) might be a prominent therapeutic agent in humans. Some potential therapeutic and biological effects on bone formation and bone density, Alzheimer disease, immunodeficiency, skin, hair, and nails condition, as well as on tumour growth, have already been documented and are critically discussed in the presented paper. Acid forms of ortho-silicic acid include: choline-chloride-stabilized ortho-silicic acid (ch-OSA) as a specific pharmaceutical formulation of H_4_SiO_4_, simple water soluble silicate salts such as sodium silicate (E550; Na_2_SiO_3_) or potassium silicate (E560; K_2_SiO_3_), and certain water-insoluble forms that, upon contact with stomach juice (HCl), release small, but biologically significant amounts of ortho-silicic acid. The latter involves: colloidal silicic acid (hydrated silica gel), amorphous silicon dioxide (E551), certain types of zeolites such as zeolite A (sodium aluminosilicate, E554; potassium aluminosilicate, E555; calcium aluminosilicate, E556), and the natural zeolite clinoptilolite. However, for some of the above-proposed therapeutic perspectives of both ortho-silicic acid and ortho-silicic acid -releasing derivatives, additional insights into biological mechanisms of action and larger studies on both animals and humans are required.

## Competing interest

The authors declare no conflict of interest.

## Authors’ contributions

LMJ has prepared the body of the manuscript text and figures as well as performed a general literature search in particular those related to animal studies. IC has prepared the literature and manuscript parts related to orthosilic acid and zeolites chemistry as well as interpretation of biological effects in relation to chemical properties. SKP has prepared the literature and parts of the manuscript related to biological effects of orthosilic acid and zeolites and performed the text revision. KP provided the idea for the manuscript, medical interpretation of cited studies and performed the final text revision. All authors read and approved the final manuscript.
